# More than a face: a unified theoretical perspective on nonverbal social cue processing in social anxiety

**DOI:** 10.3389/fnhum.2013.00904

**Published:** 2013-12-31

**Authors:** Eva Gilboa-Schechtman, Iris Shachar-Lavie

**Affiliations:** Department of Psychology, The Gonda Brain Science Center, Bar-Ilan UniversityRamat Gan, Israel

**Keywords:** social anxiety, non-verbal behavior, social rank, dominance, affiliation, production, expression

## Abstract

Processing of nonverbal social cues (NVSCs) is essential to interpersonal functioning and is particularly relevant to models of social anxiety. This article provides a review of the literature on NVSC processing from the perspective of social rank and affiliation biobehavioral systems (ABSs), based on functional analysis of human sociality. We examine the potential of this framework for integrating cognitive, interpersonal, and evolutionary accounts of social anxiety. We argue that NVSCs are uniquely suited to rapid and effective conveyance of emotional, motivational, and trait information and that various channels are differentially effective in transmitting such information. First, we review studies on perception of NVSCs through face, voice, and body. We begin with studies that utilized information processing or imaging paradigms to assess NVSC perception. This research demonstrated that social anxiety is associated with biased attention to, and interpretation of, emotional facial expressions (EFEs) and emotional prosody. Findings regarding body and posture remain scarce. Next, we review studies on NVSC expression, which pinpointed links between social anxiety and disturbances in eye gaze, facial expressivity, and vocal properties of spontaneous and planned speech. Again, links between social anxiety and posture were understudied. Although cognitive, interpersonal, and evolutionary theories have described different pathways to social anxiety, all three models focus on interrelations among cognition, subjective experience, and social behavior. NVSC processing and production comprise the juncture where these theories intersect. In light of the conceptualizations emerging from the review, we highlight several directions for future research including focus on NVSCs as indexing reactions to changes in belongingness and social rank, the moderating role of gender, and the therapeutic opportunities offered by embodied cognition to treat social anxiety.

## Introduction

Individuals with social anxiety disorder (SAD) suffer from significant functional impairment in multiple aspects of daily life: Their academic trajectory is frequently interrupted, they are employed below potential, and their social functioning suffers (Stein and Kean, 2000; Eng et al., 2005; Aderka et al., 2012). Individuals who are high in social anxiety perceive themselves as submissive and behave in nonassertive ways (Aderka et al., 2009; Schneier et al., 2009; Weeks et al., 2011). Moreover, social anxiety has been associated with lower reported quality of intimacy in peer, friendship, and romantic relationships (Rodebaugh, 2009; Cuming and Rapee, 2010; Weisman et al., 2011). Detailed analyzes of interpersonal behavior suggested that individuals with high social anxiety appear less confident, less affiliative, and less synchronous in social interactions than individuals with low social anxiety (e.g., Fydrich et al., 1998; Kachin et al., 2001; Alden and Taylor, 2004).

In this article we examine difficulties in interpersonal communication in social anxiety from the perspective of functional analysis of human sociality (e.g., Bugental, 2000). From this perspective, each individual participates in several evolutionarily shaped social structures. The two most prominent structures are affiliative relationships (friendship, companionship, intimacy) and hierarchical relationships (authority, social rank, social power). The need to affiliate with or belong to a social group is considered one of the central social motives across species, and basic psychological systems are hypothesized to constantly monitor for inclusionary status (e.g., Baumeister and Leary, 1995). Similarly, a need to advance in the social hierarchy and to be sensitive to threats to one’s status within a group appears to be inherited from our primate ancestors (Sapolsky, 2005). Social exclusion (i.e., ostracism or social rejection) and social submission (e.g., being defeated) threaten one’s belonging to and standing in a social group. Such events also decrease one’s chances of future social effectiveness and collaboration. In contrast, social acceptance and social ascendance increase one’s chances of social flourishing. In the following, we review some evidence that social rank and affiliation are basic psychological systems and then we examine their links with nonverbal social cue (NVSC).

### Social rank biobehavioral system (SRBS)

A distinctive feature of social species’ cooperative living is the formation of dominance hierarchies (e.g., Rowell, 1974). Conspecific members of the species face competition for resources, leading to aggressive interactions (West-Eberhard, 1979). Social hierarchies afford dominant members privileged access to food and mates, thereby conferring survival advantages. Such hierarchies are social systems containing an “implicit or explicit rank order of individuals or groups with respect to a valued social dimension” (Magee and Galinsky, 2008, p. 354). Social hierarchies contribute to stable social organization, and this stability reduces the costs of social competition for both dominant and subordinate members (e.g., Sloman and Armstrong, 2002). Possibly due to its importance for survival, a specialized biobehavioral system that monitors for social rank appears to have developed in humans and other mammals. This biobehavioral system has been called the rank regulation system (Zuroff et al., 2010), hierarchical domain (Bugental, 2000), power (Shaver et al., 2011), or dominance behavior system (Johnson et al., 2012).

The Social rank biobehavioral system (SRBS) is postulated as constantly monitoring one’s standing in relation to others and using that information to guide behavior (e.g., Silk, 2007). Neuroimaging evidence supported the role of limbic, prefrontal, and striatal pathways (Beasley et al., 2012) and possibly intraparietal sulci (Chiao et al., 2009) in human social rank processing. Moreover, individual differences in social rank were linked to neural activation of limbic and frontal pathways when viewing social information (e.g., Muscatell et al., 2012). The most frequently studied SRBS-related biochemical substrate has been testosterone (e.g., Schultheiss and Wirth, 2008). Testosterone was found to correlate with self-report, observational, and cognitive measures of dominance in men and women alike (e.g., Archer, 2006; Sellers et al., 2007); additionally, estradiol correlated with dominance in females (e.g., Stanton and Schultheiss, 2007).

SRBS appears to emerge early in the developmental sequence (Thomsen et al., 2011), to operate automatically (Moors and De Houwer, 2005; Tracy et al., 2013) and fluently (Zitek and Tiedens, 2012), and to be specifically attuned to certain nonverbal signals such as gaze, voice, gestures, and postures (Wolff and Puts, 2010; Terburg et al., 2012; Tracy et al., 2013). Thus, SRBS is postulated to organize and orchestrate individuals’ responses to changes in social standing.

### Affiliation biobehavioral system (ABS)

Over the course of human evolutionary history, members of the same species depended on each other for survival. In humans and other mammals, individuals who could gather support from their social surroundings gained access to more resources and therefore increased their chances of survival and reproduction. Like the dominance system, affiliative system is another basic biobehavioral system which continuously monitors for inclusionary status (Baumeister and Leary, 1995). Indeed, failing to satisfy the need to belong was found to activate neural circuits that partially overlap those of physical pain (e.g., Eisenberger et al., 2003; Dewall et al., 2010). Specifically, social exclusion was associated with greater activity in dorsal anterior cingulate cortex (dACC) and anterior insula. Again, individual differences in sensitivity to social exclusion (such as rejection sensitivity and attachment anxiety) were associated with greater responses in these regions (Linnen et al., 2012). Oxytocin and vassopressin, neuropeptides germane to affiliative behavior, may be implicated in the regulation of interpersonal stress (Taylor et al., 2000) and as affecting prosocial behavior (Poulin et al., 2012). Relatedly, intranasal administration of oxytocin reduced distress following social rejection in women who endorsed emotional coping strategies (Cardoso et al., 2012). Moreover, oxytocin administration increased prosocial behavior in women with a history of positive parenting (Riem et al., 2013). ABS emerges early in the developmental sequence (Feldman, 2012), operates automatically (Lakin et al., 2008), and is attuned to nonverbal signals of touch, gaze, and vocalization (Guastella et al., 2008; Dunbar and Abra, 2010; Farley et al., 2013).

Some convergence exists at the conceptual level between the close social bonds system and the belongingness system. In general, the jury is still out whether these two systems are best conceptualized as distinct (e.g., Panksepp and Watt, 2011), or form a single coherent unit (e.g., Feldman, 2012). For purposes of this review we focus on their similarities and view them as one. Thus, we see the ABS as organizing and orchestrating individuals’ responses to opportunities to, and ruptures in, social bonds.

### Social rank, affiliation, and nonverbal social cues (NVSCs)

Consistent evidence showed that social rank is more frequently expressed through nonverbal than verbal cues (e.g., Argyle et al., 1970; Mehrabian, 1970; Mignault and Chaudhuri, 2003). Moreover, when both verbal and nonverbal cues of social rank were present, nonverbal cues were more likely to influence observers’ judgments than verbal ones (e.g., Argyle et al., 1970; Jacob et al., 2012). Similarly, affection is also frequently expressed via nonverbal cues such as touch, vocalization, and gaze (e.g., Feldman and Eidelman, 2007; App et al., 2012; Farley et al., 2013). Indeed, people communicate emotions through multiple nonverbal channels such as face, body, voice, and touch (e.g., Buck, 1984).

Communication efficacy has been shown to vary across nonverbal channels in accruing information about likability, dominance, and trustworthiness (e.g., Zuckerman and Driver, 1989; Hall et al., 2005; Todorov et al., 2009, 2013). Researchers posited that information encoded in postures and voices is transmitted more effectively to larger audiences across longer distances, such as to one’s social group or across social groups, whereas information encoded in face and touch is more effectively transmitted to proximate others (Tracy and Robins, 2004; App et al., 2012). Correspondingly, social status emotions (such as pride or shame) were shown to be communicated more effectively through the body than through face or touch (App et al., 2012). Moreover, vocal information was found to effectively communicate dominance (Wolff and Puts, 2010), and research on animal and human behavior alike has documented strong links between expansive body postures and trait and state dominance (Weisfeld and Beresford, 1982; Ellyson and Steve, 1985; Hall et al., 2005). Indeed, Stanton et al. (2010) have postulated that the meaning of nonverbal signals as motivated and rewarding for the sender and the perceiver, respectively. Altogether, different lines of research converge in suggesting that: (a) NVSCs are uniquely suited to rapid, effective transmission of emotional, motivational, and trait information and (b) various channels differ in their effectiveness for transmitting this information.

### Social anxiety and nonverbal social cues (NVSCs)

To date, the main nonverbal signal examined in the social anxiety context was emotional facial expression (EFE; see Staugaard, 2010 for reviews). There are sound theoretical reasons to focus on EFEs. First, facial affect is instrumental in social development, emotion regulation, and social functioning (e.g., Leppänen and Hietanen, 2001). Second, facial affect is processed by specialized networks within a particular circuit of brain structures, some of which function abnormally in social anxiety (Nakao et al., 2011). Third, as argued by Bistricky et al. (2011), direct gaze can initiate automatic self-referent processing and self-relative-to-other processing (e.g., “Is his response to me unfavorable?”), and these processes are known to be problematic in social anxiety. Fourth, facial affect represents particularly salient information in close interpersonal situations (App et al., 2012).

Other NVSCs share several important characteristics with EFEs. First, NVSCs are mostly involuntary. As a result, they are more likely to serve as “honest signals” of individuals’ internal emotions or attitudes toward an interaction partner than more strategically controlled verbal content. Correspondingly, social information processing should be better attuned to NVSCs than to verbally communicated interpersonal information (e.g., Gotlib et al., 2004). Thus, NVSCs may offer more “privileged” access to individuals’ formation of approach and avoidance tendencies than would verbal expressions. Second, inasmuch as emotions are preparatory states for action, perceiving NVSCs should prime an immediate social reaction (Frijda, 1986). Indeed, some suggested that NVSCs may constitute a more evocative medium of transmitting emotions than verbal information (Buck and Vanlear, 2002). Third, given the primacy of NVSCs in the developmental sequence, they likely form the foundation for affective sensitivity and regulation (Cozolino, 2002). The preverbal foundation of implicit affective memories has been assumed to form a lasting basis for self and other schemas (e.g., Bistricky et al., 2011).

Although examinations of EFE processing have extended and deepened understanding of social anxiety’s basic processes and biases, much may be learned from expanding the framework in two ways. First, the inclusion of new signals and modalities—especially posture and voice—may elucidate how socially anxious individuals process interpersonal information and engage in impression formation and revision. In the following, we seek to summarize the rather disperse literature on NVSC processing in social anxiety, supplementing it with the burgeoning literature on voice and posture processing among nonclinical populations.

Second, we seek to expand the established framework by highlighting the important yet relatively understudied area of NVSC expression. Thus far, the vast majority of studies have examined biases in perception of NVSCs. However, the expression (or production) of those cues is central to interpersonal behavior and may provide an unbiased measure of the expresser’s emotional states and interpersonal tendencies and abilities. Moreover, expression of these cues may, in itself, affect the expresser’s cognitive and emotional states, thereby attenuating or intensifying those states (e.g., Carney et al., 2010). Consequently, we will review literature on facial, vocal, and postural expression in social anxiety and nonclinical populations.

### Organization of the review

This review has three broad goals. First, we aim to systematically review empirical studies examining NVSC perception in social anxiety. We focus mostly on research that employed information-processing approaches while incorporating pertinent cognitive neuroscience findings. Specifically, we review findings on perception of faces (including gaze orientation), voices, and bodies (postures and gestures). Second, we aim to review literature on NVSC production. Here we concentrate on mimicry of facial expression, eye gaze, vocal productions, and adoption of posture and body movements. Due to the paucity of research on some of these topics, we will draw on research with normal populations. Third, we aim to integrate these findings with the three prominent theories of social anxiety, and to formulate new testable hypotheses that may contribute to better understanding of this condition’s basic underlying mechanisms.

## Perception of nonverbal social cues (NVSCs)

Theoretical accounts converge in suggesting that misinterpretations of neutral or affiliative social signals as threatening are likely to deepen distress and contribute to the maintenance of SAD (e.g., Clark and Wells, 1995; Rapee and Heimberg, 1997; Gilbert, 2001; Alden and Taylor, 2004; Hofmann et al., 2004). Consequently, enhanced understanding of factors that influence biased or inaccurate interpretations of NVSC signals may help formulate a more complete, accurate model of SAD. We review available evidence of biases in processing of cues from faces, voices, and bodies. For each channel, we first review the perception of emotional stimuli (i.e., emotional facial expression, prosody, emotional gestures), followed by a review of impression formation from stable cues (e.g., facial features, basic vocal and body characteristics) and integrated nonverbal representations.

### Faces

#### Emotion facial expression (EFE)

We first review studies focusing on EFEs with direct gaze and then studies involving variations in gaze direction.

##### Attention

In a comprehensive review, Staugaard (2010) concluded that biased processing of threatening vs. neutral or smiling EFEs tended to emerge mostly under conditions of brief exposure and to disappear when exposure time increased. Staugaard’s review, which included mostly studies of EFE processing in non-stressful conditions, yielded only elusive differences between individuals with high vs. low social anxiety in attentional processing of threatening EFEs. Overall, eye-tracking studies found that social anxiety tends to correlate with biased attention to threatening as well as smiling EFEs. In fact, several studies found that socially anxious individuals exhibited reduced total fixation time to all emotional stimuli (e.g., Chen et al., 2012). Moreover, although highly socially anxious individuals revealed difficulty disengaging from threatening faces (e.g., Buckner et al., 2010; Moriya and Tanno, 2011; Schofield et al., 2012), they also exhibited slower attentional disengagement from smiling, as compared to neutral, faces (Gilboa-Schechtman et al., 1999). However, using a continuous flickering paradigm, Wieser et al. (2011) found that angry compared to smiling and neutral expressions were associated with greater electrocortical facilitation over visual areas in individuals with high social anxiety, but not in those with low social anxiety.

Kolassa and Miltner (2006) found that angry expressions during an emotion identification task elicited enhanced right temporoparietal N170 in individuals with SAD (Kolassa and Miltner, 2006) and in subclinically socially anxious individuals (Mühlberger et al., 2009). The research team also reported generally enhanced P100 amplitudes both in individuals with SAD and with other anxiety disorders (Kolassa et al., 2007). However, amplitudes of the N170 component and later event related potentials (ERP) components did not differ between SAD, spider phobic, and control participants when schematic emotional faces were presented (Kolassa et al., 2007) or with morphed expressions (Kolassa et al., 2009). Recently, Peschard et al. (2013) identified an enhanced P1 component in processing emotional and neutral faces in social anxiety. Combined, these data suggest that social anxiety may be related to a specific temporal pattern in processing naturalistic facial expressions.

Using fMRI, several studies employing block-designs found enhanced amygdala reactivity to angry faces (Stein, 2002; Straube et al., 2005; Phan et al., 2006; Evans et al., 2008), neutral faces (Birbaumer et al., 1998; Veit et al., 2002), and happy faces (Straube et al., 2005), pointing to hyperactivation of this area in social anxiety for all emotional expressions. Furthermore, researchers suggested that amygdala activation in highly socially anxious individuals may depend more on EFE intensity than on the particular emotion expressed (Yoon et al., 2007).

Altogether, these studies suggest that individuals high in social anxiety exhibit enhanced processing of all facial expressions in some tasks, disengagement difficulties from both threatening and smiling expression (as compared to neutral expressions) on other tasks, and enhanced vigilance/reactivity to angry expression on select tasks.

##### Interpretation and evaluation

Many studies found no association between social anxiety and labeling accuracy in emotional labeling tasks, especially when participants had unlimited time for completion (e.g., Joormann and Gotlib, 2006; Arrais et al., 2010; Campbell et al., 2009; Heuer et al., 2010). Similarly, several rating studies did not identify differences between individuals with high vs. low social anxiety in evaluating single discrete EFEs (Stein et al., 2002) or mixed displays of smiling, neutral, and angry expressions (Gilboa-Schechtman et al., 2005; Lange et al., 2011). Other studies that examined response latencies to morphed or degraded presentations of emotional expressions typically showed that social anxiety correlated with a lower threshold for identifying angry expressions (e.g., Joormann and Gotlib, 2006; Gilboa-Schechtman et al., 2008). Recently, Arrais et al. (2010) reported that, compared to women low in social anxiety, women high in social anxiety (but not men) required less emotional information to identify smiling, sad, and fearful expressions. Interestingly, when time constraints were introduced in labeling studies, biased interpretation/evaluation of EFEs emerged (e.g., Heuer et al., 2010). In addition, Heuer et al. (2007) found that, as compared to low socially anxious individuals, high socially anxious individuals showed stronger avoidance tendencies of smiling and angry faces, while no such differences with respect to neutral faces were identified. Combined, these studies suggest that persons with high social anxiety tend to exhibit more negative evaluations of ambiguous or smiling EFEs, either on implicit evaluation tasks, or under conditions of high task difficulty. Additionally, when rating studies required participants to engage in impression formation or in predicting emotional impact of possible interactions, persons with high social anxiety rated smiling faces as less approachable and angry faces as more costly (e.g., Campbell et al., 2009; Douilliez et al., 2012).

##### Memory

The social anxiety literature is mixed regarding memory biases for experimentally presented stimuli. Some studies documented enhanced processing of threatening stimuli (e.g., Foa et al., 2000), whereas others found no such biases (e.g., Rapee et al., 1994; Coles and Heimberg, 2002; Rinck and Becker, 2005 for reviews). Other studies supported the erosion of positive memory biases in SAD (Liang et al., 2011). In general, however, the support is rather modest for negative memory bias or diminished positive bias in SAD using EFEs.

#### Gaze direction

Eye gaze plays an essential role in social interactions, as averted gaze may relay various social intentions such as submission (Mazur and Booth, 1998), disinterest (Itier and Batty, 2009), or even rejection (e.g., Wirth et al., 2010). Generally, direct gaze (vs. averted) was linked with observers’ higher levels of physiological arousal (i.e., skin conductance), an effect enhanced for smiling faces (Pönkänen and Hietanen, 2012). In another study, participants rated averted gaze as less pleasant than direct gaze (Schmitz et al., 2012). In a virtual reality study, women high in social anxiety exhibited greater increase in avoidance while responding to male avatars when avatars had direct gaze than averted gaze while women with low social anxiety did not exhibit this pattern (Wieser et al., 2010). Similarly, high but not low social anxiety in women was linked with greater heart rate acceleration in response to direct, as compared to averted gaze (Wieser et al., 2009). Highly socially anxious individuals demonstrated avoidance of angry facial expressions but only when gaze was direct (vs. averted); however, they exhibited avoidance of smiling expressions regardless of gaze direction (Roelofs et al., 2010). Furthermore, an fMRI study confirmed preferential activation of brain areas related to fear response when SAD patients viewed direct gaze vs. averted gaze (Schneier et al., 2009).

It appears that sensitivity to EFEs may be modulated by gaze direction. Overall, direct (rather than averted) gaze results in higher physiological and neurological responsiveness as well as more pronounced avoidance among individuals high on social anxiety than those low in social anxiety. This sensitivity appears to be accentuated by emotional expressions; yet, more studies are needed to understand the nature of expression-gaze interaction. For example, depending on the emotional expression, direct gaze may suggest aggressive/dominant intent (when paired with angry or even neutral facial expressions) or affiliative intent (when paired with smiling).

To summarize, the EFE literature suggests that in no-stress conditions social anxiety is associated with generalized reactivity to emotional and neutral faces alike. In addition, in some but not all tasks, social anxiety is associated with selective processing of threatening EFEs, and this bias appears to be modulated by the direction of the targets’ gaze. Moreover, smiling facial expressions sometimes elicit reactions similar to those elicited by threatening EFEs. The latter finding highlights the complexity of examining facial expressions because they might simultaneously connote dominance and affiliation (Knutson, 1996). Thus, smiles could be negatively interpreted as threatening by virtue of their association with dominance. Alternatively, affiliative signals may invoke expectations of reciprocity, triggering self-evaluative concerns, which in turn can lead to fear and avoidance. The literature may benefit from better understanding how EFE processing is affected by contextual variations (such as gaze, head tilt, or gender) as well as by the perceiver’s motivational states.

### Voice

Vocal information is an important channel for assessing an interaction partner’s emotional states, intentions, and trait predispositions (Scherer, 1981; Siegman, 1987). Voices are processed early (e.g., Sauter and Eimer, 2010) and automatically (Donohue et al., 2012). Given the importance of vocal communication to the interpretation of social messages, it is surprising that only a handful of studies examined biased processing of vocal information in social anxiety. Existing studies involving social anxiety focus on labeling of, and brain responses to, emotional prosody of various utterances.

In the first study of its kind, Quadflieg et al. (2007) asked individuals with SAD and control participants to label the emotions of pseudo-words pronounced in angry, sad, happy, fearful, disgusted, or neutral tones by male and female actors. Compared to controls, individuals with SAD were more likely to label utterances as fearful or sad. Importantly, like in the evaluation of facial expressions, no group differences emerged regarding valence and arousal ratings for any of the emotional utterances.

In a follow-up study, individuals with SAD and matched controls labeled the emotion or gender of words pronounced with angry or neutral prosody by male and female actors during fMRI scanning (Quadflieg et al., 2008). Angry prosody elicited stronger brain activation than neutral prosody in limbic (insula, amygdala) as well as cortical areas for all participants. Importantly, compared to controls, individuals with SAD had increased activation in the right orbitofrontal cortex in response to angry vs. neutral voices under both task conditions. These results again substantiate findings for EFEs. McClure and Nowicki (2001) examined interpretation of EFE and vocal prosody in children who were high or low on social anxiety. Social anxiety was significantly associated with greater confusion in emotional labeling of vocal cues, where the high anxiety group was more likely than their less anxious peers to confuse other children’s sad and fearful voices.

Taken together, the study of vocal prosody suggests that social anxiety appears to affect the interpretation of prosody characteristics. However, to the best of our knowledge, no studies to date examined attention to, or memory for, prosodic information. Moreover, biases in interpreting affectionate prosody remain to be explored.

### Body and posture

In the last decade, research on facial and vocal displays of emotions has been augmented by the study of postural expressions (e.g., Tracy and Robins, 2004; App et al., 2011; Dael et al., 2012). Indeed, posture appears particularly important for portraying and recognizing affective states. Posture and associated body language serve as a rich source of information for revealing others’ goals, intentions, and emotions. These signals are visible and interpretable from a distance, do not require close contact, and allow simultaneous transmission of information to multiple individuals (e.g., Reed et al., 2003, 2006). The ability to engage in configural body processing seems to develop as early as 3 months of age (Gliga and Dehaene-Lambertz, 2005). Fearful, angry, and happy postures can be processed without visual awareness (Stienen and de Gelder, 2011). In addition, postures also transmit signals pertaining to individuals’ social rank or status (e.g., Shariff and Tracy, 2009). For example, pride is cross-culturally recognized and easily distinguished from other similar emotions (e.g., happiness; Tracy and Robins, 2007). Recently, Rule et al. (2012) found that perceivers’ judgments of posture dominance were significantly more accurate than chance guessing for exposures as brief as 40 ms, with no significant increase in accuracy given additional viewing time.

Few studies have examined processing of postural or any bodily information in social anxiety. Pitterman and Nowicki (2004) found that adults’ correct labeling of standing postures (depicting happiness, fear, anger, and sadness) correlated negatively with fear of negative evaluation. In addition, compared to children with attention disorders, children high in social anxiety made more errors identifying angry posture in adult posers (Walker et al., 2011). De Gelder and her colleagues examined how negative affectivity and social inhibition associate with the processing of threatening body expressions (fear and anger; Kret et al., 2011). They found that negative affectivity correlated with deactivation of the core emotion system (e.g., amygdala, right insula), whereas social inhibition correlated with a tendency to activate a broad cortical network (e.g., orbitofrontal cortex). Although tentative, these results suggest that social anxiety may be associated with enhanced processing of threatening or dominant postures.

### Stable features of faces, voices, and bodies

Another line of research explores the perception of stable characteristics (such as facial symmetry, vocal characteristics, and body size) as indicative of various psychological features (Tinlin et al., 2013). Substantial evidence indicates that people instantly form impressions from facial characteristics (e.g., Bar et al., 2006) and that these impressions affect important decisions (e.g., Olivola and Todorov, 2010). Regarding faces, individuals’ facial maturity was linked to their perception as dominant. When baby-faced targets were paired with submissive information (congruent condition), they were better remembered than when they were paired with dominant (non-congruent) information (Cassidy et al., 2012). In addition, increase in men’s face-width-to-height ratio was linked to their faces being perceived as more aggressive (Carré et al., 2010). Thus, stable facial characteristics appear influential in judgments of people as dominant and/or affiliative.

Studies with non-clinical populations have examined the influence of stable auditory characteristics of voice—especially pitch—on trait judgments. Specifically, male participants made judgments of likely physical aggressiveness based on vocal recordings of men reading a sentence, which were raised or lowered in both fundamental frequency (F0) and formant dispersion (df; Wolff and Puts, 2010). Raised vocal masculinity (lowered F0 or df) yielded higher dominance ratings. Moreover, raters with either high or low levels of testosterone rated other men as more dominant than raters with mean levels of testosterone.

Stable characteristics of body may also influence perceptions of power and dominance. Height is an important factor, as taller individuals were perceived as more dominant (Adams, 1980; Melamed, 1992; Young and French, 1998), even when females were evaluated (Boyson and Butler, 1999).

Recent studies from our group have shown that social anxiety is associated with biased impression formation and impression revision from trait descriptions (Aderka et al., 2013; Haker et al., 2013). Yet, to date, studies have yet to assess the role of social anxiety in the interpretation of static characteristics of the faces, voices, or bodies. Such an endeavor may enrich understanding of how social anxiety affects person perception.

### Integrated nonverbal representations

Integrating information from face, voice, and body plays a crucial role in human ability to understand social interactions. Although distinct, these different sources of information are normally processed in parallel, and, when synchronous, facilitate social comprehension. In contrast, the study of channel incongruity may enable understanding of the relative diagnostic value of each component of person perception. For example, participants predicted that the face would be most influential on perceptions of winning and losing emotions; however, the body was the best diagnostic feature (Aviezer et al., 2012). Rule et al. (2012) also found that face-body gestalt increased accuracy in perceiving dominance expressions from bodies and faces. Van den Stock et al. (2007) demonstrated that body postures conveying emotions influenced recognition of facial expressions and tones of voice. These findings emphasize the importance of emotional whole-body expressions, as well as the combination of NVSCs in everyday settings. With respect to social anxiety, the examination of conflicting cues—such as those connoting threat in one channel and affiliativeness in another—may elucidate the relative diagnosticity of each information type for socially anxious individuals.

### Evaluative summary of nonverbal social cue (NVSC) perception

Taken together, the research reviewed above suggests that individuals with high social anxiety are more likely than peers with low social anxiety to misinterpret EFEs and vocal expressions of emotions, although results pinpointing threatening misinterpretations were not as robust as theoretically expected. While pertinent studies on body posture are scarce, it appears likely that socially anxious individuals may be also biased in processing these stimuli. Examination of socially anxious individuals’ perceptions of nonverbal representations of social status cues is important, given that social status is central to evolutionary (e.g., Gilbert and Trower, 2001), interpersonal (Alden and Taylor, 2004), and cognitive (e.g., Clark and Wells, 1995) accounts of social anxiety.

## Production of nonverbal social cues (NVSCs)

Existing research has focused on NVSC perception, but multiple considerations indicate the need to extend investigation to the domain of NVSC production. First, examination of expressive indices of emotion can substantially complement subjective reports by linking research on adult humans to research on infants (Cappella, 1981) and non-human primates (e.g., Geerts and Brüne, 2009). Second, expressivity was pinpointed as an important clue for judging cooperation (e.g., Boone and Buck, 2003; Schug et al., 2010) and thus may direct an interaction’s outcome. Third, based on embodied cognition accounts, researchers found that expressive behaviors lead to cognitive change (Briñol et al., 2011). These behavior-cognition and cognition-behavior links may generate either a self-enforcing positive or negative cycle, again influencing the course of interactions. Finally, NVSC production was shown to predict the long-term course of depression following treatment (Bos et al., 2006, 2007). Thus, NVSC production may possibly also be used to predict treatment outcomes in social anxiety.

### Faces

#### Facial mimicry

Mimicry has been conceptualized as affiliative social behavior where one emulates another person’s nonverbal actions (Lakin and Chartrand, 2003). By and large, mimicry was found to generate positive social feelings (Chartrand and Bargh, 1999). For example, priming individuals with prosocial (as opposed to antagonistic) goals generated more mimicking behavior (Leighton et al., 2010). Also, a priori manipulation of liking for another increased mimicry (Stel et al., 2010). Furthermore, automatic mimicry responses increased following a social exclusion situation, possibly because the threat of exclusion promoted affiliative motivations and actions (Lakin et al., 2008).

Importantly, mimicry depends on an interaction’s nature; competitive interactions seem to elicit less facial mimicry than collaborative interactions (Likowski et al., 2011). Lower mimicry in competitive situations may be conceptualized as complementary behavior, where dominant displays (e.g., anger) elicit submissive reactions (e.g., fear). Interestingly, mimicry not only affects this social signal’s recipient but also it’s expresser: women were slower to recognize the affective valence of briefly displayed facial expressions when constrained from mimicking them (Stel and van Knippenberg, 2008). This effect was attributed to the fact that facial constraints hinder women’s capacity to empathize.

Only a handful of studies explored automatic facial mimicry in social anxiety. In the first study to address this issue, Dimberg (1997) found that women high in public speaking fearfulness reacted with more frowning to angry faces than did women low in public speaking fearfulness, and women low in public speaking fearfulness exhibited more reactivity to happy facial expression than did women high in public speaking fearfulness (Dimberg, 1997). Vrana and Gross (2004) found that individuals high in public speaking fear exhibited more frowning in response to angry, neutral and happy facial expressions than did individuals low in speech fearfulness. Finally, Dimberg and Thunberg (2007) found that the individuals in a high public speaking fear group exhibited greater negative facial reactivity when responding to angry vs. happy faces, and greater positive emotional reactivity when responding to happy, as compared to angry faces. Given the recent emphasis on importance of mimicry for the establishment of rapport and liking on the one hand, and the deficits in affiliative behavior in social anxiety on the other (e.g., Alden and Taylor, 2011), more studies examining mimicry in social anxiety are needed.

#### Facial expressivity: voluntary displays of affect

Emotional expressivity is the extent to which an individual manifests emotional impulses behaviorally (Gross and John, 1997). Emotional expressivity was linked to increased positive affect (Gross and John, 1995; Burgin et al., 2012), whereas suppressing emotional displays was shown to decrease positive affect (Gross, 1998). Emotional expressivity was also linked to better social functioning (Burgin et al., 2012), cooperativeness (Schug et al., 2010), and agreeableness (Gross and John, 1995). Moreover, expressivity is beneficial not only when it concerns positive emotions. Feinberg et al. (2012) found that people who expressed embarrassment were judged as more prosocial. In terms of dominance, Hall et al. (2005) found that more gazing, nodding, and smiling and a generally more expressive face were associated with high interpersonal power.

Social anxiety is linked to lower levels of emotional expressivity (Kashdan and Breen, 2008), and is found to be mediated by beliefs that overt emotional expression is negative (Spokas et al., 2009). Moreover, Kashdan et al. (2011) found that the negative correlation between social anxiety and positive affect was mediated by the tendency to suppress emotional displays.

In sum, expressivity emerges as an important nonverbal variable in social interactions, signaling both dominant and affiliative propensities. Social anxiety is associated with lower levels of expressivity, constituting a potential deficiency in both social domains.

#### Eye gaze

Robust evidence suggests that avoidance of eye contact may be a central characteristic of SAD (e.g., Schneier et al., 2011). Specifically, fear and avoidance of eye contact consistently correlate with overall severity of social anxiety (e.g., Safren et al., 1999; Baker and Edelmann, 2002; Stein et al., 2004), and were recently found to decrease with successful treatment (e.g., Schneier et al., 2011). Studies relying on independent observers’ judgments revealed that individuals with SAD made less eye contact during social interactions than individuals low in social anxiety (Baker and Edelmann, 2002; Voncken and Bögels, 2008). Advancements in eye-tracking methodology have permitted in-depth examination of this feature of socially anxious behavior: Individuals with SAD showed fewer gaze fixations on the eyes when facial expressions were presented for relatively long time intervals (e.g., Horley et al., 2004; Moukheiber et al., 2010, 2012; but see also Wieser et al., 2009).

Theoretically, gaze avoidance has been linked to submissive behavior in a variety of species (e.g., Mazur and Booth, 1998). Inasmuch as human social anxiety has been postulated as related to such submissive behaviors (e.g., Gilbert, 2001), perhaps gaze processing in social anxiety holds promise for creating a neurobiological marker of this disorder.

### Voice

Individuals with SAD are often concerned about showing auditory signs of anxiety during social performance (e.g., Hirsch and Clark, 2007). Early studies seeking to examine the veracity of these concerns tested the vocal performance of socially anxious individuals in interpersonal situations. Such highly socially anxious individuals were often rated by others as less competent in their vocal communication compared to controls (e.g., Borkovec et al., 1974; Fydrich et al., 1998; see review in Baker and Edelmann, 2002). Further studies highlighted relations between social anxiety and temporal features of spontaneous speech. For example, highly socially anxious and clinically distressed individuals with SAD paused more often and for longer durations, demonstrated slower speech rate, and evidenced restricted verbal output (e.g., Borkovec et al., 1973; Lewin et al., 1996; Hofmann et al., 1997).

In a pioneering study, Laukka et al. (2008) used acoustic analysis to explore the effect of social anxiety on objectively defined auditory parameters. Public speech samples of individuals with SAD were recorded preceding and following pharmacological intervention. Participants who reported lower anxiety following treatment demonstrated post-treatment decreases in mean F0 (subjectively perceived as pitch) and decreased proportions of silent pauses. In another study, Weeks et al. (2011) placed socially anxious men in a competitive interaction with another man over the positive attention of a female peer. Consistent with evolutionary predictions, highly socially anxious men manifested increased mean F0, whereas men low in social anxiety showed the opposite trend. Recently, Weeks et al. (2012) also compared acoustic characteristics of public speaking between a group with SAD and non-anxious controls. Males with SAD evidenced greater F0 in comparison to non-anxious individuals across both studies. For females, the inverse correlation between social anxiety and F0 was significant only when examined in patients with generalized SAD, and in response to in vivo social exposures. Importantly, gender-specific thresholds for mean F0 demonstrated excellent differentiation between patients with generalized SAD and non-anxious controls.

Galili et al. (2013) examined college students’ vocal characteristics as a function of social anxiety by asking participants to record neutral, command, and request sentences and then analyzing these utterances’ acoustic properties (mF0, intensity, speech rate, speech fluency). Social anxiety was associated with higher mF0 in men and women and with lesser vocal intensity in men. Moreover, compared to neutral sentences, social anxiety was associated with lesser increase of vocal intensity in command utterances, and with greater decrease of vocal intensity in request utterances. In men but not women, social anxiety also correlated with slower speech rate in request sentences. Taken together, these results pinpoint F0 as a promising biobehavioral marker of social anxiety (at least in men). Moreover, vocal intensity, speech rate, and speech fluency also seem likely to be affected by social anxiety in socially stressful situations.

Other nonverbal parameters of speech such as volubility (time spent talking; Mast, 2002; Brescoll, 2012), successful interruptions (Farley, 2008), vocal expressivity (e.g., Dunbar and Abra, 2010), initiation of speech acts, and laughter (Gifford and Hine, 1994) have been linked with high interpersonal power. Likewise, volubility was the best predictor of observer-rated social performance in an interaction task (Stevens et al., 2010). Similar findings emerged in an impromptu speech task, where socially phobic patients exhibited significantly less volubility than individuals low in social anxiety (Beidel et al., 2010).

In sum, different aspects of acoustic performance appear to link significantly with social anxiety. Acoustic parameters of speech associate closely with manifestations of power and social rank in both humans (e.g., Dunbar and Abra, 2010) and other mammals (e.g., Koren et al., 2008; Laporte and Zuberbühler, 2010). Altogether, these findings point to the possible diagnosticity of this expressive behavior.

### Posture and body movement

Body posture and movement are important indicators of powerful behavior. Specifically, power is associated with more bodily openness, more erect or tense posture, and more body or leg shifts (Hall et al., 2005). Recently, Weeks et al. (2011) found that high social anxiety levels were associated with slumped and closed posture when interacting with a male competitor, whereas low social anxiety was associated with expansive posture. Other studies on observer ratings found that socially phobic individuals exhibited more bodily discomfort during social or performance tasks, such as rigidness and fidgeting (Voncken and Bögels, 2008; Heiser et al., 2009). Finally, measurement of observers’ head movements showed that, when viewing an avatar’s whole body, observers with high social anxiety mimicked the avatar significantly less than individuals with low social anxiety (Vrijsen et al., 2010).

### Evaluative summary of nonverbal social cue (NVSC) production

Vocal and bodily behaviors appear linked to social anxiety and to expression of social rank. Facial expression and eye gaze seem linked both to expression of dominance (e.g., anger, contempt) and expression of affiliation (e.g., smiles). Existing research indicates that highly socially anxious individuals, especially males, will more likely exhibit submissive behaviors than individuals with low social anxiety.

Production of non-affiliative or submissive NVSCs is likely to deepen people’s sense of disconnection and/or ineptitude either by directly influencing cognitions (e.g., Briñol et al., 2011) or through standards of culturally-appropriate behavioral norms (e.g., one should look one’s offender in the eye, rather than lowering one’s head; people should look others in the eye and smile). Thus, gaining more complete, complex understanding of NVSC production may advance more nuanced conceptualizations of interpersonal and situational factors that influence self-evaluations in social anxiety.

## Integration of theoretical approaches to social anxiety

### Nonverbal social cues (NVSCs) at the epicenter of social encounters

Independent theoretical contingents have identified different factors contributing to the maintenance of social anxiety: cognitions (e.g., Clark and Wells, 1995; Rapee and Heimberg, 1997; Gilbert, 2001; Hofmann et al., 2004), interpersonal factors (e.g., Alden and Taylor, 2004), or evolutionary pressures (Gilbert, 2001). Recent attempts were made to integrate these theoretical literatures. Our lab proposed a cognitive-evolutionary model (e.g., Aderka et al., 2013; Galili et al., 2013; Haker et al., 2013; Gilboa-Schechtman et al., in press), and several other researchers (Levinson et al., 2011; Taylor and Alden, 2011; Moscovitch et al., 2012) argued for a cognitive-interpersonal hypothesis. To varying degrees, the cognitive, interpersonal, and evolutionary models implicate abnormal social behavior and cognition in the onset and maintenance of social anxiety. Indeed, the three theories tend to be more complementary than contradictory. Importantly, all three postulate that socially anxious individuals are biased when perceiving social signals such as facial expressions, and because those signals guide interpersonal interactions, that bias leads to important outcomes. Interpersonal theories, evolutionary theories, and cognitive embodiment accounts also highlight the importance of the production of interpersonal signals (e.g., smiles, powerful postures) for the coordination of social interactions. Thus, the processing and production of NVSC is the juncture at which these theories intersect.

The evolutionary/interpersonal dimension of social rank and affiliation refine and sharpen the cognitive categories of threat and safety. Consider, for example, a case of an expanded pride posture (Tracy and Robins, 2004). While such posture may not represent social threat, it does connote an attempt to ascend in social rank. According to our account, such cues are likely to be selectively processed by individuals high in social anxiety. Conversely, the cognitive-embodiment account help explain the link between intra- and inter-personal mechanisms. For example, an assertive voice tone engenders a sense of self-assurance, which, in turn, elicits a complimentary (e.g., obliging, submissive) response from the interaction partner (e.g., Tiedens and Fragale, 2003). Moreover, we claim that the maintaining factors in social anxiety rely not only on the way individuals’ process NVSC (the self-as-receiver of information) but also on the way individuals’ *express* socially relevant attitudes and behavior (the self-as-an-agent).

### Reactivity to social stress in social anxiety: nonverbal social cue (NVSC) and beyond

Most theoretical models of SAD consider heightened sensitivity to, enhanced responsivity to, and impaired regulation in the face of social threat to be at the epicenter of this condition (e.g., Clark and Wells, 1995; Rapee and Heimberg, 1997; Gilbert and Trower, 2001; Hofmann et al., 2004). Individuals with SAD reported heightened emotional reactions to both positive and negative social events (e.g., Gilboa-Schechtman et al., 2000). Experimental interpersonal manipulation studies consistently found that individuals with high social anxiety or SAD reported more intense, persistent negative affect in anticipation of, and following, various social challenges such as public speaking (e.g., Rapee and Lim, 1992; Ly and Roelofs, 2009), social ostracism (Oaten et al., 2008) and social success (Wallace and Alden, 1997) compared to individuals with low social anxiety.

Research has yet to examine the impact of social anxiety on the sensitivity to and bias in interpreting NVSCs following social stress. As our review suggests, two types of distinct social events appear crucial for such future examination: events indicating changes in belongingness (e.g., social exclusion, gaining social favor) and events indicating changes in social rank (e.g., defeat, victory). Examination of sensitivity to diverse stressors is pivotal, as sensitivity to interpersonal cues signaling affiliation or social rank is essential for smooth navigation in the interpersonal world, especially when one seeks to recover from a social misfortune or to capitalize on social success. For example, following exclusion, a failure to correctly identify potential friends or allies (vs. foes) is likely to hamper social reintegration, thereby increasing the threat of additional rejection. Similarly, the failure to assert oneself once a social group observes one’s positive qualities or deeds may hamper one’s chances of advancing in the social hierarchy. Finally, the mediating effects of social anxiety on the tendency to interpret NVSCs as affiliative or dominant may elucidate the causal structure of attunement difficulties in this population.

### Future directions

The present perspective calls for several directions of future research. First, the current unified perspective accentuates the need to study gender differences in perceiving and perhaps even more so in expressing NVSCs. Men and women face somewhat different threats in navigating groups; women face greater risk for exclusion, whereas men face greater risk for physical defeat (e.g., Archer, 2004; Benenson et al., 2011). Thus, we can expect gender to moderate the relations between social anxiety and perception of exclusion and dominance signals, where women are more sensitive to exclusion and men to dominance. Moreover, based on the tend-and-befriend theory of women’s response to social threat, females may be expected to engage in more affiliative gestures following exclusion or defeat, whereas men may be more likely to express signals of deference or submissiveness (Taylor, 2006).

Second, existing evidence points to the possibility that embodiment of powerful NVSCs may lead to congruent changes in cognitions and cognitive processing and, conversely, the adoption of submissive NVSCs may deepen existing cognitive biases. For example, recent studies by Galinsky et al. (2006) suggested that merely thinking about powerful experiences enhances performance (Lammers et al., 2013). Moreover, both power postures and power roles were shown to reduce interpersonal fearfulness and increase approach behaviors; yet, power postures were more effective (Huang and Galinsky, 2010). Similarly, recent studies found that pitch does not merely correlate with social rank—it also instills subjective feelings of dominance and cognitive correlates of power (Stel et al., 2012). Altogether, these studies suggest reciprocal patterns of influence between NVSCs and cognition, with dominant expressions enhancing sense of power and submissive expressions decreasing it. Thus, loss of felt power may result in fewer displays of expressive behaviors in general and greater displays of submissive or appeasement behaviors (Stel et al., 2012). Such changes in expressive production may foster self-perceptions of powerlessness. These findings underscore an intriguing possibility for embodiment-focused cognitive interventions, such that socially anxious individuals may engage in training to implicitly or explicitly affect their sense of power or dominance.

Finally, research on NVSCs may provide converging evidence regarding the brain circuits engaged in social anxiety. For example, if processing of several types of NVSCs is found to involve overlapping circuitry (amygdala, insula, DLPFC), such brain areas may be associated with a special significance for social anxiety. More broadly, focusing on the social rank and affiliation systems may offer a new and helpful conceptual framework to examine brain mechanisms previously labeled approach-related and avoidance-related (e.g., Quirin et al., 2013; Terburg and van Honk, 2013).

## Conclusion

NVSC are signals with long evolutionary history. As such they figured prominently in cognitive, interpersonal, and evolutionary accounts. In the present review we emphasized the links between biased processing of NVSC and social anxiety, highlighting the tendency of the socially anxious to be sensitive to social status cues. We also emphasized that production of non-affiliative or submissive NVSCs is likely to deepen a sense of disconnection and ineptitude. Given these propensities, socially anxious individuals might perceive and remember the social world as a hierarchical and competitive arena. These perceptions, in turn, may lead to a persistent sense of incompetence and inferiority (Gilbert et al., 2009). In the review we emphasized the interplay between perception and expression of NVSC as concomitant with social anxiety; future work would profit from examining the causal status of NVSC as contributing to the onset and maintenance of this condition.

## Conflict of interest statement

The authors declare that the research was conducted in the absence of any commercial or financial relationships that could be construed as a potential conflict of interest.
